# Associations between demographic and disease related factors and anxiety and depression among adolescents with chronic kidney disease

**DOI:** 10.3389/fped.2025.1616765

**Published:** 2025-08-22

**Authors:** Changchang Bai, Mingjie Wei, Shuli Huang, Junmei Fu, Guigui Ning, Wensheng Zhai

**Affiliations:** ^1^ College of Nursing, Henan University of Chinese Medicine, Zhengzhou, China; ^2^ Pediatric Hospital of the First Affiliated Hospital of Henan University of Traditional Chinese Medicine, Zhengzhou, China

**Keywords:** adolescents, anxiety, depression, risk factors, chronic kidney disease

## Abstract

**Objective:**

To investigate the current status of comorbid anxiety and depression in adolescents with chronic kidney disease and to analyze their associated factors.

**Methods:**

105 adolescents with chronic kidney disease, hospitalized in the First Affiliated Hospital of Henan University of Traditional Chinese Medicine from May 2022 to May 2023, were selected through convenience sampling. A general information questionnaire, anxiety self-assessment scale, and depression self-assessment scale were used. Factors associated with anxiety and depression were analyzed using univariate analysis, and significant variables underwent multinomial logistic regression.

**Results:**

Among the 105 adolescents, 11 cases (10.5%) had anxiety alone, 6 cases (5.7%) had depression alone, and 26 cases (24.8%) presented both anxiety and depression. Multinomia logistic regression analysis revealed that the illness duration (>12 months) (OR = 34.114), duration of hormone treatment (≥12 months) (OR = 37.585), duration of immunotherapy (≥12 months) (OR = 12.700), and OR values greater than 1 indicate that illness duration (>12 months), duration of hormone treatment (≥12 months), and the duration of immunotherapy (≥12 months) are independent associated factors for nanxiety and depression.

**Conclusion:**

In this cross-sectional cohort study, the illness duration, duration of hormone treatment and duration of immunotherapy are independent associated factors for anxiety and depression. Psychological screening should be prioritized for adolescents with these clinical features, and relevant measures should be taken to reduce the incidence of anxiety and depression in adolescents with CKD. Future longitudinal studies could begin by elucidating whether modifying treatment can reduce mental health risks, taking into account potential bidirectional effects between disease control and mental health.

## 1 Introduction

Chronic kidney disease (CKD) is a progressive disease with irreversible changes in kidney structure and function caused by various factors (1). It is characterized by lengthy diagnostic and therapeutic cycles, being difficult to cure, and often leading to psychological abnormalities in patients, with depression and anxiety being common psychological problems. Recent years show CKD prevalence in children ranging from 14.9/1,000,000 to 118.8/1,000,000 (2), becoming a serious public health concern as pediatric kidney disease incidence increases annually (3–5). Adolescence is a critical period for children's growth and development. It is not only a peak period for physical development, but also a critical period for the gradual formation of personality, cognition, and worldview. However, adolescents are sensitive to external stimuli and have relatively low tolerance for setbacks and failures (6). Adolescents with CKD face developmental challenges while managing disease-related stressors, potentially leading to psychological and social dysfunction (7). Studies indicate that the global prevalence of anxiety disorders in children and adolescents is 2,235.1 per 100,000 (8), with an anxiety detection rate of 34.6% in adolescents, depressed mood at 28% (9–11), and depression detection rates exceeding 19.7% (9, 12). Current research on anxiety and depression in CKD primarily focuses on adults, with fewer studies examining adolescents (1, 13). There are already many validated anxiety and depression assessment scales, but these tools have mainly been validated in healthy or generally ill adolescents, with a lack of research in adolescents with CKD (14, 15). General scales may not capture stressors unique to adolescents with CKD, such as fear of dialysis/transplantation, body image concerns, academic/social disruption, and concerns about the future, and therefore may not fully reflect their psychological distress. Therefore, this study primarily investigates the current status of anxiety, depression, and comorbidity among adolescents with CKD, and explores their influencing factors. The aim is to provide a basis for the clinical prevention and treatment of anxiety, depression, and comorbidity among adolescents with chronic kidney disease, and to provide a reference for the formulation of psychological intervention treatment plans for adolescents with CKD in the future. The findings are as follows:

## 2 Information and methods

### 2.1 Study subjects

1. Inclusion criteria:
  a. Children with CKD who have been regularly followed up for ≥6 months at our hospital's Pediatric Nephrology Center;
  b. Meet the diagnostic criteria of CKD in Chinese and Western medicine, and the diagnosis is clear;
  c. Age ≥12 years old and ≤19 years old;
  d. Duration of the disease ≥6 months;
  e. Children and their family members signed an informed consent form and voluntarily enrolled in the study.
2. Exclusion criteria:
  a. Children who do not meet the diagnostic criteria of CKD in Chinese and Western medicine;
  b. Children with CKD whose family members do not cooperate well and cannot maintain clinic attendance;
  c. Children with a history of anxiety and depression.

A total of 354 patients who met the criteria were consecutively enrolled, and 123 agreed to participate. A total of 18 participants withdrew for various reasons, resulting in a final study sample of 105 participants.

### 2.2 Investigative instruments

#### 2.2.1 General information survey

A self-designed general information survey form was used, which included the following items: sex (male, female), age, only child (no, yes), educational Background (Primary school, junior high school, high school, university, college, other), family residence (countryside, cities and towns, municipalities), family status (single-parent family, two-parent family, stepfamily), residence during school (attend a day school, homestay, suspend schooling), parents' educational level (junior high school or below, high school/secondary school, specialized training school, Undergraduate and above), illness duration (6–12 months, more than one year), Type of disease (Nephrotic Syndrome, Purpura Nephritis, Lupus Nephritis, Glomerulonephritis, Renal Failure, Membranous Nephropathy, IgA Nephropathy), Duration of hormone treatment (<3 months, 3–5 months, 6–11 months, ≥12 months), Duration of immunosuppressive therapy (<3 months, 3–5 months, 6–11 months, ≥12 months, not have), Duration of kidney replacement therapy (<3 months, ≥3 months, not have), disease follow-up period (2 weeks, 1 month, 3 months, 6 months). If the child's parents are unaware of certain personal information, the child may seek assistance in completing the form.

#### 2.2.2 Anxiety and depression scales

The Self-Administered Scale of Anxiety (SAS) and Depression Self-Assessment Scale (SDS) were developed by Dr. Zung in 1971 and 1965, respectively, to provide a brief, efficient, self-rated screening tool for anxiety and depressive symptoms in primary care and psychiatric clinical practice. They were designed to rapidly identify individuals who may need further professional evaluation (16, 17). Since their release, SAS and SDS have been widely used worldwide, including in China, especially in large-scale epidemiological investigations, clinical studies, and preliminary screening, favored for their ease of operation, ease of understanding, and implementation. Each scale comprises 20 self-rating scales, addressing the respondent's experience in the preceding week. Examples include “I feel more nervous and anxious than usual” and “I feel depressed and low in mood,” with response options rarely, sometimes, often, and continuously, scored 1–4 (total score 20–80 points). Total scores are converted to index scores using the formula: index = (raw score/80) × 100, and the index score ranges from 25 to 100, Based on clinical experience and early research data, Zung proposed the cut-off criteria of index scores (SAS ≥ 50, SDS ≥ 53) in the original literature to indicate the level of significant anxiety/depression symptoms. Multiple studies have evaluated the reliability of the SAS/SDS. In the general population and in a variety of clinical samples, the Cronbach's α coefficients are typically reported for the SAS between 0.70 and 0.85. The Cronbach's α coefficient for SDS showed a similar level, typically between 0.79 and 0.88 (18, 19).

### 2.3 Survey method

A questionnaire-based anonymous survey was used. Hospitalized children who met the inclusion criteria received instructions on questionnaire completion from the investigator on the second or third day of hospitalization. Parents were instructed to minimize their participation, and the children completed the survey independently.

### 2.4 Pre-survey

Using random sampling, a pre-survey was conducted with 15 children of different ages who met the inclusion criteria. The survey revealed that the average completion time was 119–183 s. Children could understand and complete the questionnaire independently, while family members demonstrated high cooperation and minimal participation.

### 2.5 Data collection and quality control

1. An anonymous QR code was generated using the Questionnaire Star platform, which was set to not collect any personal information. Patients scanned the code themselves and completed the questionnaire independently in a relatively private space, ensuring anonymity from the outset;
2. Researchers present are strictly limited to explaining the process, clarifying the meaning of questions (not answer choices), addressing operational queries, monitoring progress, and reminding parents not to participate. However, if parental personal information is involved, the primary caregiver may provide relevant answers to the patient, and researchers are not medical staff from the same ward;
3. Researchers cannot view real-time answers and cannot link answers to patients before submission;
4. After submission, success is confirmed via platform notifications or backend status (without reviewing answer content). The platform's mandatory response feature and on-site supervision ensure questionnaire completeness;
5. Data is double-checked after export.
6. A total of 105 questionnaires were distributed, with 105 valid responses collected, resulting in a 100% valid response rate.

### 2.6 Ethics consideration

The study protocols received informed consent from patients and their families and were approved by the hospital's Ethics Committee.

### 2.7 Statistical methods

SPSS 22.0 software performed the statistical analysis. Variables underwent normality testing, with normally distributed data expressed as *x* ± *s*, Non-normal distributions are represented by the median and quartiles. Qualitative data were described by frequency and percentage. Independent sample t-tests compared two groups, while ANOVA or rank-sum tests compared multiple groups to identify variables affecting anxiety and depression in adolescents with CKD. Multinomial logistic regression analyzed significant variables, with *P* < 0.05 considered statistically significant.

## 3 Results

### 3.1 General information of survey respondents

Table 1 describes the demographic and chronic kidney disease-related characteristics of the sample. A total of 105 valid questionnaires were obtained, with a response rate of 100%. Among the study participants, 62 children (59.0%) did not meet the threshold for anxiety or depression, 11 children (10.5%) had anxiety alone, 6 children (5.7%) had only depression, and 26 children (24.8%) had both anxiety and depression. Among them, 37 children had anxiety symptoms, with a detection rate of 35.2%; 32 children had depression symptoms, with a depression detection rate of 30.5%. See Table 1.

**Table 1:** Results of general information survey of 105 participants.

Participant characteristic	Categories	*N*	%
Age	>13 and ≤15	45	42.9
	>15 and ≤19	60	57.1
Sex	Male	69	65.7
	Female	36	34.3
Whether or not you are an only child	Yes	12	11.4
	No	93	88.6
Schooling	Primary school	34	32.4
	Junior high school	34	32.4
	High school	17	16.2
	College	7	6.7
	Other	13	12.4
Residence of the family	Countryside	65	61.9
	Cities and towns	25	23.8
	Municipalities	15	14.3
Family situation	Single parent family	3	2.9
	Two-parent family	98	93.3
	Stepfamily	4	3.8
Residence during the school year	Attend a day school	62	59.0
	Homestay	25	23.8
	Suspend schooling	18	17.1
Parents' educational level	Junior high school and below	65	61.9
	High school/secondary school	25	23.8
	Specialized training school	5	4.8
	Undergraduate and above	10	9.5
Illness duration	6–12 months	40	38.1
	More than one year	65	61.9
Type of disease	Nephrotic syndrome (medicine)	57	54.3
	Purpura fulminans	30	28.6
	lupus erythematosus (kidney disease)	5	4.8
	Glomerulonephritis	2	1.9
	Renal failure	2	1.9
	Membranous nephropathy	3	2.9
	IGA nephropathy	6	5.7
Duration of hormone treatment	<3 months	22	21
	3–5 months	20	19
	6–11 months	24	22.9
	≥12 months	39	37.1
Duration of immunotherapy	None	14	13.3
	<3 months	37	35.2
	≥3 months and <6 months	10	9.5
	6–11 months	12	11.4
	≥12 months	32	30.5
Duration of kidney replacement therapy	none	58	55.2
	<3 months	34	32.4
	≥3 months	13	12.4
Disease follow-up period	2 weeks	29	27.6
	1 month	62	59.0
	3 months	14	13.3
	Half a year	0	0

### 3.2 Univariate analysis of anxiety and depression scores among adolescents with chronic kidney disease with different demographic characteristics

Schooling, parents' educational level, illness duration, duration of hormone treatment, duration of immunotherapy, and duration of kidney replacement were significant factors associated with anxiety symptoms (*P* < 0.05); schooling, illness duration, duration of hormone treatment, duration of immunotherapy were significant factors associated with depression symptoms (*P* < 0.05) see Table 2.

**Table 2:** Univariate analysis of anxiety and depression scores in adolescents with chronic kidney disease (*n* = 105, `*x* ± *s*, points).

Variables	Categories	*N*	SAS score			SDS score		
			$\bar{x}\pm s$	*t*/*F* value	*P*-value	$\bar{x}\pm s$	*t*/*F* value	*P*-value
Age	>13 and ≤15	45	43.11 ± 10.28	−0.690	0.492	45.69 ± 9.34	−0.808	0.421
	>15	60	44.54 ± 10.69			47.29 ± 10.51		
Sex	Male	69	44.02 ± 10.23	0.125	0.900	46.09 ± 10.00	−0.736	0.667
	Female	36	43.75 ± 11.12			47.60 ± 10.14		
Only child or not	Yes	12	42.60 ± 13.13	−0.463	0.644	46.04 ± 10.55	−0.207	0.836
	No	93	44.10 ± 10.17			46.68 ± 9.99		
Schooling	Primary school	34	42.79 ± 10.08	4.412	0.003*	44.89 ± 10.05	4.570	0.002*
	Junior high school	34	44.82 ± 9.59			47.02 ± 8.23		
	High school	17	51.03 ± 9.09			54.26 ± 9.40		
	College	7	42.14 ± 15.01			45.89 ± 13.07		
	Other	13	36.25 ± 7.47			40.38 ± 8.16		
Residence of the family	Countryside	65	44.88 ± 9.62	0.967	0.384	48.06 ± 9.80	1.852	0.162
	Townships	25	43.30 ± 11.91			43.95 ± 10.33		
	Municipalities	15	40.83 ± 11.67			44.75 ± 9.82		
Family situation	Single parent family	3	46.25 ± 13.92	2.787	0.066	50.42 ± 5.20	1.054	0.325
	Two-parent family	98	43.38 ± 10.37			46.24 ± 10.17		
	Stepfamily	4	55.63 ± 2.98			52.81 ± 6.32		
Schooling and residence	Attend a day school	62	44.07 ± 10.86	0.052	0.949	46.29 ± 9.96	0.091	0.913
	Homestay	25	44.10 ± 10.09			47.30 ± 9.06		
	Suspend schooling	18	43.19 ± 10.27			46.74 ± 11.81		
Parents' educational level	Junior high school and below	65	46.38 ± 10.48	3.779	0.013*	48.37 ± 9.82	2.013	0.117
	High school/secondary school	25	39.85 ± 8.645			43.70 ± 9.43		
	Three-year college	5	36.00 ± 11.97			41.00 ± 10.36		
	Undergraduate and above	10	42.13 ± 9.93			45.25 ± 11.01		
Illness duration	6–12 months	40	37.59 ± 8.14	−5.494	0.000^**^	41.59 ± 7.78	−4.361	0.000^**^
	More than 1 year	65	47.83 ± 9.90			49.69 ± 10.03		
Type of disease	Nephrotic syndrome (medicine)	57	44.56 ± 11.35	0.366	0.899	45.56 ± 10.55	1.280	0.273
	Purpura fulminans	30	43.00 ± 9.43			45.00 ± 8.03		
	Lupus nephropathy	5	43.50 ± 11.50			54.50 ± 14.51		
	Glomerulonephritis	2	35.00 ± 5.30			35.63 ± 2.56		
	Renal failure	2	46.25 ± 5.30			51.88 ± 2.56		
	Membranous nephropathy	3	47.08 ± 12.83			50.83 ± 4.73		
	IGA nephropathy	6	43.54 ± 9.82			48.33 ± 11.45		
Duration of hormone treatment	<3 months	22	41.59 ± 9.21	4.307	0.007*	43.47 ± 7.74	4.368	0.006*
	3–5 months	20	40.94 ± 8.31			43.81 ± 7.70		
	6–11 months	24	41.15 ± 9.99			44.74 ± 11.37		
	≥12 months	39	48.49 ± 11.18			50.96 ± 10.08		
Duration of immunotherapy	None	14	45.71 ± 9.01	9.897	0.000^**^	43.04 ± 11.38	8.205	0.000^**^
	<3 months	37	37.23 ± 8.12			41.93 ± 8.02		
	3–5 months	10	44.38 ± 9.85			44.75 ± 11.08		
	6–11 months	12	43.65 ± 9.47			48.44 ± 6.03		
	≥12 months	32	50.86 ± 9.69			53.48 ± 8.69		
Duration of kidney replacement	None	58	46.08 ± 9.98	3.553	0.032*	48.47 ± 9.22	2.304	0.105
	<3 months	34	40.18 ± 10.88			44.23 ± 10.27		
	≥3 months	13	44.13 ± 9.63			44.52 ± 11.72		
Disease follow-up period	2 weeks	29	43.58 ± 9.88	2.123	0.125	45.43 ± 9.41	1.557	0.216
	1 month	62	45.22 ± 11.18			47.92 ± 10.18		
	3 months	14	38.93 ± 6.95			43.21 ± 10.03		
	Half a year	0	-			-		

* *P* < 0.05.

** *P* < 0.01.

**Table 3:** Multinomial regression of associations between disease-related characteristics and anxiety and depression Among adolescents with chronic kidney disease.

Disease-related characteristic	Anxiety		Depression		Depression G anxiety	
	OR^a^ [95% CI^b^]	AOR^c^ [95%CI]	OR^a^ [95% CI^b^]	AOR^c^ [95%CI]	OR^a^ [95% CI^b^]	AOR^c^ [95%CI]
Illness duration						
6–12 months	Ref	Ref	Ref	Ref	Ref	Ref
>12 months	14.861* [1.609–137.237]	12.740* [1.221–132.972]	9.605 [0.866–106.550]	7.707 [0.595–99.882]	17.607* [1.368–226.610]	34.114* [1.697–685.617]
Duration of hormone treatment						
<3 months	Ref	Ref	Ref	Ref	Ref	Ref
3–5 months	1.419 [0.137–14.721]	1.691 [0.144–19.848]	0.534 [0.030–9.410]	0.675 [0.036–12.497]	1.270 [0.047–34.493]	0.635 [0.019–20.779]
6–11 months	1.471 [0.183–11.849]	1.865 [0.198–17.534]	0.304 [0.018–5.116]	0.469 [0.025–8.753]	4.428 [0.260–75.434]	3.158 [0.162–61.461]
≥12 months	1.846 [0.194–17.57]	3.125 [0.272–35.831]	0.808 [0.065–9.992]	1.270 [0.090–17.854]	42.210* [3.171–561.908]	37.585* [2.575–548.513]
Duration of immunotherapy						
None	Ref	Ref	Ref	Ref	Ref	Ref
<3 months	0.981 [0.076–12.599]	1.050 [0.079–14.008]	0.330 [0.016–6.740]	0.371 [0.018–7.525]	0.165 [0.009–3.076]	0.217 [0.009–5.465]
3–5 months	5.408 [0.351–83.411]	6.973 [0.356–136.425]	2.064 [0.090–47.366]	2.905 [0.098–85.678]	0.576 [0.026–12.898]	0.928 [0.030–28.311]
6–11 months	3.718 [0.219–63.081]	9.502 [0.444–203.406]	3.730 [0.225–61.782]	7.593 [0.356–161.928]	0.720 [0.034–15.370]	1.014 [0.032–31.672]
≥12 months	2.086 [0.136–32.028]	1.985 [0.115–34.269]	0.736 [0.031–17.662]	0.717 [0.028–18.668]	10.925* [1.184–100.782]	12.700* [1.133–142.417]
Duration of kidney replacement						
None	Ref	Ref	Ref	Ref	Ref	Ref
<3 months	2.581 [0.434–15.366]	5.602 [0.732–42.868]	1.250 [0.128–12.227]	2.384 [0.199–28.606]	2.162 [0.243–19.205]	1.634 [0.148–18.000]
3–5 months	1.029 [0.115–9.201]	1.436 [0.133–15.469]	0.999 [0.065–15.272]	1.206 [0.068–21.403]	0.985 [0.100–9.699]	1.005 [0.081–12.510]

* *P* < 0.05.

^a^ OR, crude Odds Ratio.

^b^ CI, Confidence Interval.

^c^ AOR, adjusted Odds Ratio (adjusted for patient's schooling and primary caregiver's literacy level).

### 3.3 Multinomial logistic analysis of factors influencing anxiety and depression levels

Taking anxiety, depression, anxiety and depression as dependent variables, the statistically significant variables in the univariate analysis, namely disease-related variables (illness duration, duration of hormone treatment, duration of immunotherapy, and use of renal replacement therapy), and demographic variables (schooling, parents' educational level) as independent variables, were used for multinomial logistic regression analysis. The results showed that:

In isolated anxiety, patients with a disease duration of >12 months had an odds ratio for anxiety that was nearly 13 times higher than that of patients with a disease duration of 6–12 months (reference group). In isolated depression, the odds of having depression among patients with a disease duration of >12 months were nearly 8 times higher than those with a disease duration of 6–12 months (reference group), but this difference was not statistically significant; in anxiety and depression, the odds of having anxiety and depression among patients with a disease duration of >12 months were nearly 34.5 times higher than those with a disease duration of 6–12 months (reference group).

In isolated anxiety, patients who used hormones for 3–5 months, 6–11 months, and ≥12 months had odds of anxiety that were nearly 2-fold, 2-fold, and 3.5-fold higher than those who used hormones for less than 3 months (reference group), but these differences were not statistically significant. In isolated depression, patients who used hormones for 3–5 months, 6–11 months, and ≥12 months had odds of depression that were approximately 0.7 times, 0.5 times, and 1.5 times those of patients who used hormones for less than 3 months (reference group), respectively, but these differences were not statistically significant; In anxiety and depression, the odds of having anxiety and depression among patients with hormone use duration ≥12 months were approximately 38 times higher than those with hormone use duration <3 months (reference group); Patients who used hormones for 3–5 months and 6–11 months had odds of anxiety and depression that were approximately 0.7 times and 3.5 times those of the reference group (less than 3 months), respectively, but these differences were not statistically significant.

In isolated anxiety, patients who used immunosuppressants for less than 3 months, 3–5 months, 6–11 months, and ≥12 months had odds ratios of approximately 1-fold, 7-fold, 9.5-fold, and 2-fold, respectively, compared to those who used immunosuppressants for other durations (reference group), but these differences were not statistically significant; In isolated depression, patients who used immunosuppressants for less than 3 months, 3–5 months, 6–11 months, and ≥12 months had odds ratios of approximately 0.4, 3, 7.5, and 0.7, respectively, compared to those who used immunosuppressants for none (reference group), but these differences were not statistically significant. In anxiety and depression, the odds ratio for patients with immunosuppressant use duration ≥12 months was approximately 13 times that of the reference group (other durations). For patients with anxiety and depression who used immunosuppressants for less than 3 months, 3–5 months, and 6–11 months, the odds were approximately 0.2 times, 1 times, and 1 times, respectively, compared to those who used immunosuppressants for other durations (reference group), but these differences were not statistically significant.

In isolated anxiety, the odds of anxiety in patients with renal replacement therapy duration of less than 3 months and 3–5 months were nearly 6 times and 1.5 times higher, respectively, than in the reference group (other durations), but these differences were not statistically significant; In isolated depression, the odds of having depression during the first 3 months and 3–5 months of renal replacement therapy were nearly 2.5 times and 1.5 times higher, respectively, compared to other durations of renal replacement therapy (reference group), but these differences were not statistically significant; In anxiety and depression, the odds of anxiety and depression in patients with a renal replacement therapy duration of less than 3 months and 3–5 months were approximately 2 times and 1.5 times higher, respectively, compared to the reference group (other durations), but these differences were not statistically significant (see Table 3).

## 4 Discussion

Due to the complexity of CKD's etiology, the extended treatment period, and the challenges in diagnosis and treatment, patients frequently experience anxiety, depression, and other negative emotions that adversely affect therapeutic outcomes (20). This study's results revealed that Among 105 adolescents with CKD, 37 (35.2%) had anxiety symptoms and 32 (30.5%) had depression symptoms,the illness duration, duration of hormone and duration of immunotherapy have factors associated with anxiety and depression. These findings align with both international and domestic studies (21, 22).

Studies have shown (23) that care levels help reduce anxiety and depression in adolescents, and parents' education and cultural background may influence their understanding and support of their child's illness. Parents with higher education levels better analyze information, show greater initiative, and pay more attention to their children's illnesses. They also demonstrate higher acceptance of healthcare professionals' health promotion guidance (24). Conversely, parents with lower education levels may lack disease knowledge and coping strategies, potentially increasing their children's adverse emotions. In this study, 61.9% of caregivers had junior high school or lower education levels. Caregivers with lower educational attainment may have a less complete understanding of disease progression and prognosis (25). This limited understanding has been associated with caregiver distress and adjustment difficulties in other studies (26), which could, in turn, potentially contribute to higher levels of anxietyand depression observed in their children in this study.

CKD is a complex condition characterized by prolonged treatment cycles and a high risk of recurrence. According to Kogon et al. (27), the timing of CKD diagnosis may correlate with anxiety and depression, suggesting that patients' burden and stress increase over time, leading to these conditions. CKD's long-term progressive nature, regular follow-ups, disease progression, and continuous medication use affect children's adaptability and interpersonal skills. According to Nair's study, school-age adolescents with CKD often cannot attend school normally due to recurrent episodes of the disease (28). Frequent follow-up visits and outpatient appointments lead to missed classes, school absences, or missed group activities, depriving adolescents of opportunities to develop essential social and academic skills the prolonged course of the disease imposes a heavy psychological burden on adolescents (29, 30). Roshan (31) found that adolescents' cognitive and personality development is not yet mature, making them more susceptible to external stimuli. Teenagers with CKD are in a crucial period of developing interpersonal communication skills and social adaptability. Due to the recurrence of the disease, they are unable to attend school regularly, and the time spent with classmates and teachers is greatly reduced. These interpersonal relationship deficiencies and limited relationships will aggravate negative emotions. Meanwhile, the age group of this study mainly consists of junior and senior high school students, who have heavy learning tasks and great learning pressure. Frequent medical visits lead to repeated absences from classes, which affects studies. As grades decline, pressure increases and learning difficulties arise, anxiety and depression symptoms may be triggered or exacerbated. Therefore, the longer the course of the illness, the more likely it is to lead to the emergence of negative emotions.

CKD is mainly treated with hormone drugs, but long-term use of hormones can cause changes in the body, thereby triggering anxiety related to appearance. According to Trautmann et al. (32), children receiving long-term glucocorticoid maintenance therapy usually have full-moon faces, buffalo backs, and hirsute skin, while adolescent children are more self-conscious and care more about their external image. Children with chronic diseases often experience side effects related to their appearance due to long-term steroid medication, leading to labels such as “sick,” “unpopular,” or “boring,” which can result in rejection, mockery, isolation, and bullying by peers (33). Additionally,in the face of changes in appearance and body shape brought about by medication, they develop more negative emotions. This is consistent with the results of this study. The children in this study are in adolescence, when psychological changes are in a more sensitive stage, and they pay more attention to their self-image. Due to the long-term use of hormonal drugs for disease treatment, appearance anxiety arising from the side effects of the drugs increases the occurrence of anxiety and depression symptoms.

CKD not only requires the use of hormones but also the periodic use of immunosuppressants. The use of immunosuppressants may lead to disorders and suppression of the immune system, increasing the risk of infections and other complications. Restrictions on social activities, sleep, and diet can easily aggravate the anxiety of children with CKD. Additionally, the long-term special treatment of these children and the protective measures required for treatment give them a sense of “disease shame.” Especially on campus, they may be ridiculed because of their disease, become alienated from their peers, and prone to depression (34). For patients with advanced CKD, renal replacement therapy, such as dialysis or kidney transplantation, may be required. This treatment process can significantly impact the child's lifestyle, self-image, and social activities, increasing the risk of anxiety and depressive symptoms. Therefore, healthcare professionals should promptly recognize these factors and take appropriate measures to help reduce anxiety and depression in adolescents with CKD and improve their quality of life and survival.

## 5 Limitations of this study

Due to the limitations of the conditions, only CKD adolescent patients attending one hospital were selected as research subjects. There may be bias in subject selection; the sample is not representative enough. The sample size included in this study was small and statistically underpowered. These limitations affect the study's conclusions. In future research, the selection scope should be expanded, and the sample size increased to improve the conclusions' accuracy. This study is investigative; future pilot studies could examine the effects of targeted nursing interventions on anxiety and depression in adolescents with CKD and develop standardized treatment plans to reduce their anxiety and depression.

## 6 Conclusions

In summary, the prevalence of comorbid anxiety and depression among CKD adolescents is high. The illness duration, duration of hormone treatment and duration of immunotherapy are independent associated factors for anxiety and depression. Clinical measures should address these factors, monitor CKD adolescents' mood changes dynamically, actively support family and school participation, assist in stress relief, and provide psychological support through active listening to reduce anxiety and depression incidence in adolescents with CKD.

## Data Availability

The original contributions presented in the study are included in the article/Supplementary Material, further inquiries can be directed to the corresponding author.
